# Cochleates Derived from *Vibrio cholerae* O1 Proteoliposomes: The Impact of Structure Transformation on Mucosal Immunisation

**DOI:** 10.1371/journal.pone.0046461

**Published:** 2012-10-12

**Authors:** Reinaldo Acevedo, Oliver Pérez, Caridad Zayas, José L. Pérez, Adriana Callicó, Bárbara Cedré, Luis García, David Mckee, Alexander B. Mullen, Valerie A. Ferro

**Affiliations:** 1 Research and Development Vice-presidency of Finlay Institute, Havana, Cuba; 2 Department of Physics, University of Strathclyde, Glasgow, United Kingdom; 3 Strathclyde Institute of Pharmacy and Biomedical Sciences, University of Strathclyde, Glasgow, United Kingdom; The Scripps Research Institute and Sorrento Therapeutics, Inc., United States of America

## Abstract

Cochleates are phospholipid-calcium precipitates derived from the interaction of anionic lipid vesicles with divalent cations. Proteoliposomes from bacteria may also be used as a source of negatively charged components, to induce calcium-cochleate formation. In this study, proteoliposomes from *V. cholerae* O1 (PLc) (sized 160.7±1.6 nm) were transformed into larger (16.3±4.6 µm) cochleate-like structures (named Adjuvant Finlay Cochleate 2, AFCo2) and evaluated by electron microscopy (EM). Measurements from transmission EM (TEM) showed the structures had a similar size to that previously reported using light microscopy, while observations from scanning electron microscopy (SEM) indicated that the structures were multilayered and of cochleate-like formation. The edges of the AFCo2 structures appeared to have spaces that allowed penetration of negative stain or Ovalbumin labeled with Texas Red (OVA-TR) observed by epi-fluorescence microscopy. In addition, freeze fracture electron microscopy confirmed that the AFCo2 structures consisted of multiple overlapping layers, which corresponds to previous descriptions of cochleates. TEM also showed that small vesicles co-existed with the larger cochleate structures, and *in vitro* treatment with a calcium chelator caused the AFCo2 to unfold and reassemble into small proteoliposome-like structures. Using OVA as a model antigen, we demonstrated the potential loading capacity of a heterologous antigen and *in vivo* studies showed that with simple admixing and administration via intragastric and intranasal routes AFCo2 provided enhanced adjuvant properties compared with PLc.

## Introduction

Cochleates (Co) are phospholipid-calcium precipitates derived from the interaction of anionic lipid vesicles with divalent cations such as calcium. They have a defined multilayered structure consisting of a solid, lipid bilayer sheet rolled up in a spiral. Papahadjopoulos *et al.*, 1975 [1] first described Co as an intermediate in the preparation of large unilamellar liposomes. Since then, Co have been used to deliver proteins, peptides and DNA for vaccine applications by oral and nasal routes [2]. Co have a lot of advantages over other particulate structures such as liposomes, tubules, or ribbons because they are thermodynamically more stable and permit a high loading of components with different physicochemical properties [3]. Thus, hydrophilic and hydrophobic molecules can be accommodated between the lipid bilayers and inner hydrophobic space, respectively [3]. A novel and proprietary strategy developed by the Finlay Institute employs proteoliposomes (PL) extracted from bacteria as a source of negatively charged molecules including phospholipids, proteins, and lipopolysaccharides, to induce cochleate formation in the presence of divalent cations [4]. The Adjuvant Finlay Cochleate 1 (AFCo1) derived from *Neisseria meningitidis* serogroup B proteoliposomes (AFPL1) is more stable and immunogenic when administered via the nasal route compared with the proteoliposomes [5]. We have expanded the Co formation strategy to other microorganisms such as *Vibrio cholerae* to develop a novel mucosal formulation against this and related enteric pathogens. This was achieved by preparing proteoliposomes from *V. cholerae* O1 El Tor Ogawa, C7258 strain (called PLc) and then transforming them into new tubular structures designated AFCo2 [6].

The aim of this paper was to evaluate the molecular organization of AFCo2, using electron microscopy to gain a greater understanding of the formation of the membranes in AFCo2, to evaluate the interaction of AFCo2 with a heterologous antigen (OVA) and to assess the immune response induced when the antigen was admixed and administered via intragastric and nasal routes.

## Materials and Methods

### Dialysis rotary method for producing cochleates

AFCo2 from *V. cholerae* were obtained as described by Acevedo *et al.* (2009) [6]. Briefly, we re-suspended and adjusted PLc to 1 mg/mL (protein content) in a buffer containing 30 mmol/L tris(hydroxymethyl)aminomethane (Tris) and 1.5% (w/v) sodium deoxycholate (DOC). AFCo2 formation was performed by detergent elimination and Ca^2+^ incorporation using rotary dialysis against a wash buffer containing 30 mmol/L Tris, 100 mmol/L NaCl and 5 mmol/L CaCl_2_ at pH 7.4. Five to six washes were carried out at 2 hourly intervals. As visual precipitation occurred during the course of processing, supplementary observations were made by light microscopy using an Opton Standard 25 microscope and the size of the particles determined using a gradation scale on the ocular lens.

### Negative Staining and Transmission Electron Microscopy (TEM)

Sample preparations were carried out by Dr Laurence Tetley (Glasgow University, UK) as follows: Formvar/Carbon-coated 200 mesh copper grids were glow discharged. Cochleate samples were resuspended in 30 mM Tris buffer and dried with filter paper to a thin layer onto the hydrophilic support film. Twenty microlitres of 1% (v/v) aqueous methylamine vanadate stain (Nanovan; Nanoprobes, Stony Brook, NY, USA) was applied and excess moisture removed with filter paper. Dried samples were imaged with a LEO 912 energy filtering transmission electron microscope operating at 120 kV. Contrast enhanced, zero-loss energy filtered digital images were recorded with a 14 bit/2 K Proscan CCD camera.

### Scanning electron microscopy (SEM)

SEM was carried out as described by Qu *et al.* [7]. Briefly, samples were dissolved in the Tris-Ca wash buffer and frozen on glass coverslips in liquid propane/isopentane (3∶1 v/v), then lyophilised (overnight at −80°C) mounted on specimen stubs with conductive copper tape prior to applying a 20 nm gold/palladium coating with a Polaron SC515 sputter coater using a Peltier cold stage. SEM was performed on a JSEM 6400 instrument (JEOL Ltd, UK) with an ADDA3 digital interface and 2 K format images were recorded operating at 6 or 3 kV to enhance surface imaging.

### Freeze-fracture electron microscopy (FFEM)

Sample procedures were carried out as described by Qu *et al.* [7]. Briefly, samples were sandwiched between two copper support plates (Bal-Tec AG, Balzers, Lichtenstein) and snap-frozen by plunging them into a cryogenic mixture of propane/isopentane 3∶1 (v/v) at −190°C. These were then stored at −150°C. Fracturing was carried out at −100°C at 10^−6^ torr with the fracture face replicated with platinum/carbon (PtC, Agar Scientific, UK) and carbon coated at a 90° angle to the surface. Acetone was used to clean the replicates and they were collected onto a mesh (100 grid bars/in), dried and examined in a LEO 912 energy filtering transmission electron microscope operating at 80 kV. Contrast enhanced, zero-loss energy filtered digital images were recorded with a 14 bit/2 K Proscan CCD camera.

### Fluorescence experiments and sample preparation

AFCo2 and PLc were mixed separately with OVA labelled Texas Red (OVA-TR) (Invitrogen, Ltd. UK) for 10 min with agitation. The formulations were washed three times by ultra-centrifugation (10 000 g×1 h) using an Amicon 100 000 kDa filtration unit to eliminate unbound OVA-TR. Adsorbed OVA on the surface of the structures was removed by sonication and then washed again three times in the Amicon unit to eliminate non-adsorbed OVA-TR. The structures were observed using a Zeiss Axioplan 2 epifluorescence microscope (Carl Zeiss Jena GmbH, Jena, Germany) and imaged with a Canon 350D DSLR camera. Filtrates and pellets were analyzed with a fluorescence microplate reader (SpectraMax M5, Molecular Devices, Corp, US) at 615 nm and 596 nm. AFCo2 pellets were treated with EDTA (50 mM) as described in the next section. The loading average of OVA-TR was calculated as: OVA-TR [Pellet+Filtrate]/OVA-TR [Pellet]×100, using an OVA calibration curve.

### Observation of AFCo2 unfolding and sample preparation

EDTA solutions ranging from 5 to 50 mM were incubated with 0.05 mg/mL AFCo2. Briefly, AFCo2 samples were centrifuged at 3000 g for 5 min and the supernatant discarded. The pellet was re-suspended up to the same volume with the different EDTA concentrations. The number of unfolding structures was evaluated by light microscopy at 400× magnification using a Reichert-Jung Polyvar microscope and recorded using a JVC MicroCam camera coupled to the microscope.

### Photon correlation spectroscopy (PCS)

Particle size, polydispersion and zeta potential of the samples were analyzed using a Malvern Instruments Zetasizer Nano ZS operated at 25°C using triplicate readings and a 2 min equilibration time.

### Immunization and sample collection

Groups of female BALB/c mice (6–8 weeks old, CENPALAB, Cuba) were immunized intranasally or intragastrically with AFCo2 or PLc (100 µg per dose per mouse). These mice are an established model used in our laboratory to evaluate cochleate adjuvant activity [6]. Mice received three administrations by intragastric (i.g) gavage (200 µL) or intranasal (i.n) instillations (10 µL per nostril) once a week (0, 7 and 14). Placebo groups were also immunized with 20 µL (10 µL per nostril) of phosphate buffered saline (PBS) by the i.n route or 200 µL by the i.g route. Mucosal (saliva and faeces) and sera samples were taken at 7 and 14 days after the last immunization, respectively. Prior to immunization, samples were also collected and prepared as described previously [6]. Animals were housed at the Finlay Institute animal facility and all experiments were performed with the approval of the Finlay Institute Ethical Committee.

### Enzyme-linked immunosorbent assay (ELISA) for PLc specific or OVA specific IgA, IgG and IgG isotypes

Specific IgG, IgG1 and IgG2a anti-PLc antibodies in serum samples and IgA anti-PLc antibodies in saliva or faeces were measured by indirect ELISA using Nunc-Immuno MaxiSorp 96-well plates. Plates were coated with PLc (100 µL per well) at 5 µg/mL or OVA at 5 µg/mL (100 µL per well) in PBS buffer (0.15 mol/L, pH 7.3) at 4°C overnight, and blocked with 1% (w/v) BSA in PBS (blocking solution) for 1 h at room temperature. Serum samples were serially diluted (1∶50 to 1∶3600) and saliva (1∶2 to 1∶32) in blocking solution and incubated for 1 h at 37°C. Anti-mouse IgG or IgA peroxidase-conjugated antibodies (Sigma, USA) were added (100 µL per well) at 1∶2500 dilution in blocking solution and incubated for 1 h at 37°C. For IgG isotypes anti-mouse IgG biotinylated antibody (1∶1000) were used (Sigma, USA). Bound antibodies were detected with 100 µL per well of the substrate-chromogen mixture (o-phenylenediamine and hydrogen peroxide in citrate-phosphate buffer, pH 5). The reaction was stopped by adding 50 µL of sulphuric acid at 2 mol/L and optical density at 492 nm was measured using a microplate reader (Titertek, Multiskan Plus, Germany). All incubation steps were followed by three washes with PBS containing 0.05% Tween-20 (v/v). Positive antibody titers were defined as the highest dilution giving an OD value that was twice that of negative controls (pre-immune serum, saliva or faeces). Antibody measurements were expressed as the logarithm of the geometric mean of three different experiments ± SEM.

### Vibriocidal antibody assay

Serum vibriocidal antibodies were determined as described previously [6]. Briefly, 50 µL of two-fold dilutions of decomplemented sera in PBS were mixed with 50 µL of a *V. cholerae* VC12 (serotype Ogawa) suspension containing 10^8^ CFU/mL in PBS supplemented with 2% (w/v) guinea pig complement and placed in a 96-well microtitre plate. The mixture was incubated for 1 h at 37°C and subsequently supplemented with 100 µL of Brain Heart Infusion Broth containing 2% (w/v) dextrose and 0.02% (w/v) bromocresol purple. The plates were incubated at 37°C for 3 h. Bacterial growth was determined by a colour change of the medium, which indicated bacterial dextrose consumption. During the bactericidal reaction the serum dilution in the first column of the plate was 1∶20 and in the last one was 1∶10240. The vibriocidal antibody titer was calculated as the inverse of the highest dilution of serum causing complete inhibition of bacterial growth (no change of colour in the medium). The results were expressed as the logarithm of the geometric mean of three different experiments ± SEM.

### Statistical Analysis

All the statistical analyses were performed using GraphPad Software Inc, La Jolla CA, USA. Statistical significance of the variance between multiple groups was calculated with one-way ANOVA, followed by a Tukey's multiple comparison test (statistical significance, p<0.05). The experimental data was first subjected to a Kolmogorov-Smirnov normality test with Dallal-Wilkinson-Lillie for p-value.

## Results

### Transformation of PLc into AFCo2

**Figure 1:**
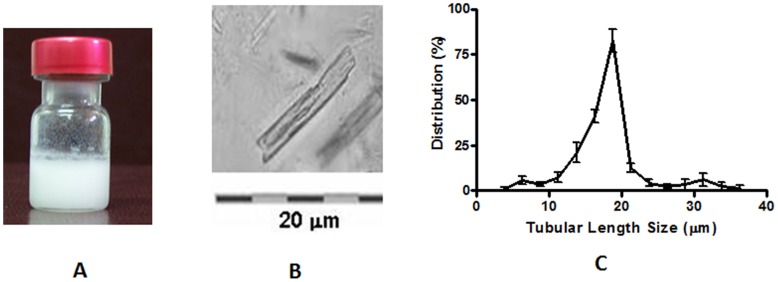
AFCo2 formation. (A) White milky suspension (AFCo2) obtained after calcium interaction with a solution of PLc, (B) AFCo2 observed by light microscopy with an Opton Standard 25 microscope (magnification ×400), (C) distribution percentage (82.6%) of AFCo2 with a length of 16.3±4.6 µm measured using a graduated scale on the ocular lens of the microscope.

PLc structures of approximately 160.7±1.6 nm in size were transformed into AFCo2 using a dialysis rotary method [6]. The process was characterized by a slow transformation in the visual appearance of the solution from a colourless solution (PLc) to a milky white suspension (Figure 1A). Figure 1B shows a micrograph of the AFCo2 observed by light microscopy and Figure 1C the size distribution, 16.3±4.6 µm, determined using a gradation scale on the microscope.

### Electron microscopy reveals cochleate structures

**Figure 2:**
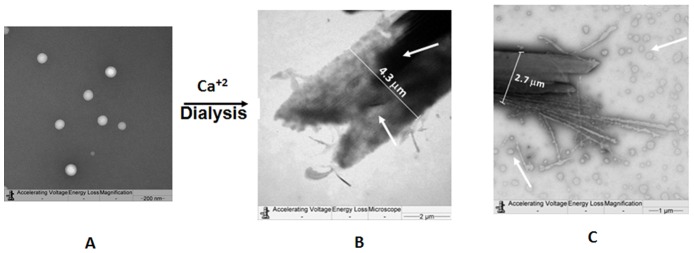
Supramolecular transformation of PLc in AFCo2, observed by TEM. (A) Negatively stained PLc (×10000 magnification), (B) AFCo2, tubular structure observed using negative stain (×1000) [arrows indicate the distribution of stain inside the AFCo2 structure], (C) AFCO2 and vesicles (arrows) co-existing (×2000).

Micrographs obtained by TEM show that the PLc (Figure 2A) changed at supramolecular level in a calcium environment and became transformed into AFCo2 structures (Figure 2B). It is noteworthy that the PLc bilayer was not permeable to negative staining, whereas the stain diffused to some extent into the AFCo2. However, this diffusion was not homogeneously distributed. These micrographs also reveal that the AFCo2 structures had widths in the range of 2 to 4.5 µm.

**Figure 3:**
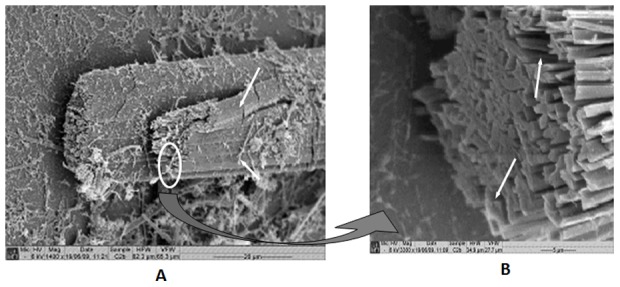
Scanning electron micrographs of AFCo2. (A) Tubular and multilamellar structures observed at ×1400 [multilamellarity is indicated by white arrows], (B) the open end of the structure magnified ×3300.

Figure 3A (SEM micrograph) shows that AFCo2 consist of multiple overlapping layers. Magnification of Figure 3B reveals hollows or pores found mainly at the edges of the structures.

**Figure 4:**
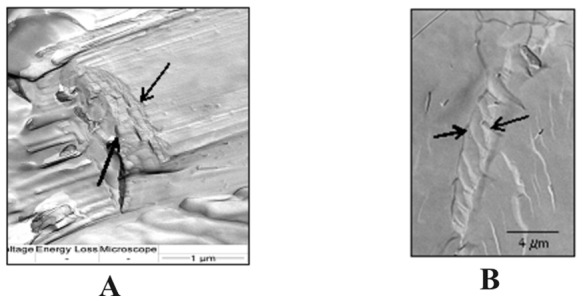
Freeze fracture electron micrographs of AFCo2. (A) Cylindrical shape of AFCo2 and black arrows point to the multiple layers forming the structure, (B) Overlapping layers can be observed with a curvature and disposition similar to the classic spiral pattern characteristic of cochleate structures.

Micrographs obtained by FFEM show that AFCo2 appear to be compact cylinders and confirm previous SEM results where multiple fractured layers are observed (Figure 4A). The typical spiral organization of the cochleates is identifiable in Figure 4B where curvature in the different layers forming the AFCo2 can be observed.

### Internalization and loading of a fluorescent probe in AFCo2

**Figure 5:**
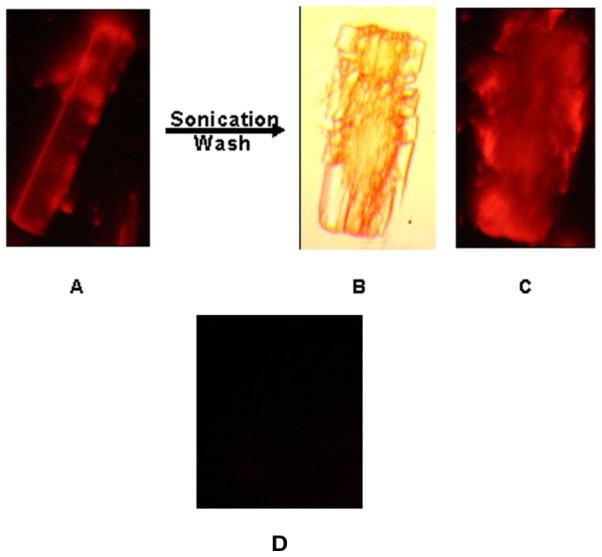
Labeling AFCo2 and PLc with OVA-TR. (A) AFCo2 admixed with OVA-TR. The images were obtained with a coupled-device camera linked to an epi-fluorescence microscope. After sonication and ultrafiltration AFCo2+OVA-TR was analyzed by light microscopy as shown in (B). The broken structure is a consequence of the sonication process. (C) The same AFCo2 structure observed by epi-fluorescence showing the distribution of the Ova-TR in AFCo2. (D) PLc+OVA-TR observed by epi-fluorescence showing a lack of signal.

In this experiment the OVA-TR was associated with AFCo2 after mixing (Figure 5A), but little or no fluorescence was observed from the sample of OVA-TR mixed with PLc (Figure 5D). Loading experiments showed 46% of OVA-TR was entrapped in the AFCo2, however less than 2% of the OVA-TR was calculated to be associated to PLc. Additionally, AFCo2 could be disrupted by sonication (Figure 5B) and any unbound OVA-TR removed by washing. Figure 5C shows that the disrupted AFCo2 still emitted an OVA-TR signal; the higher intensity coming from within the structure. Finally, zeta potential measurements revealed that the AFCo2 surface had a slightly negative charge −7.25±0.25 mV compared with PLc, which had a charge of −31.2±0.42 mV.

### Cochleates unfold into small vesicles in the presence of EDTA

**Figure 6:**
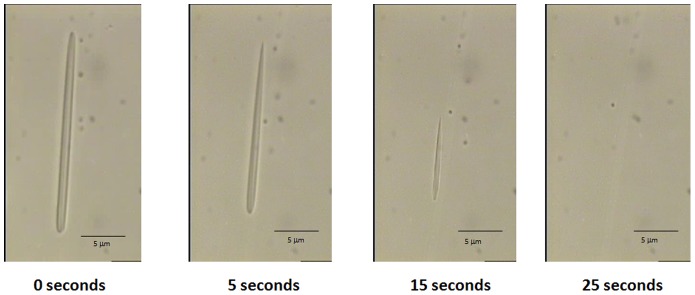
AFCo2 unfolding with EDTA. The micrographs represent stills of Video S1 (supporting information) that shows the unfolding process of AFCo2 under the action of EDTA chelating agent (25 mM). AFCo2 starts to shrinks in the first 15 seconds and completely disappears at 25 seconds of exposure with EDTA.

Different concentrations of EDTA ranging from 1 to 50 mM were used to sequestrate calcium from the AFCo2 in order to unfold the structure. Table 1 shows that the best concentrations for unfolding 50 µg (by protein content) per mL of AFCo2 were 25 and 50 mM EDTA. The measurements were obtained using light microscopy; the results show the number of particles observed in 64 fields using a Neubauer chamber. A recording was made to show a cochleate structure unfolding under EDTA action (Video S1, supporting information). Photon correlation spectroscopy analysis also revealed that EDTA treated AFCo2 re-formed into vesicles with a size about 503±8.0 nm and a polydispersion index of 0.32±0.04. Figure 6 shows the unfolding effect of 25 mM EDTA in AFCo2. The tubular structure begins to shrink in the first 20 seconds before finally disappearing.

**Table 1:** AFCo2 unfolding in the presence of EDTA.

EDTA [mM]	Quantity of unfolded particles
1	+++
5	++
10	+/−
25	-
50	-

AFCo2 was resuspended at a final protein concentration of 0.05 mg/mL with buffer containing Tris 30 mM and EDTA ranging from 1 to 50 mM. The results show the number of particles observed in 64 fields in a Neubauer chamber using light microscopy. EDTA concentrations from 25 to 50 mM were suitable for completely unfolding the AFCo2.

+++ more than 100 particles observed per view, ++ 40–80 particles, −/+ 10–40 particles, - no particles.

### Evaluation of immune response induced by mucosal administration of AFCo2

Female BALB/c mice were immunised via the i.g or i.n route with 100 µg of AFCo2 or PLc using a three dose schedule (0, 7 and 14 days). Since PLc are transformed into AFCo2, both structures contain the same *V. cholerae* antigens. ELISAs were carried out to detect specific anti-PLc antibodies in mucosal (saliva and faeces) and systemic (sera) collected samples. Table 2 shows that mice were only responsive at mucosal level with AFCo2 administered by the i.g route. Intragastrically immunised mice with PLc did not elicit any antibody response. However, mice immunised with AFCo2 or PLc by the i.n route elicited similar immune response (IgG and vibriocidal activity) in sera (p>0.05). The IgG2a/IgG1 response in mice immunised with AFCo2 or PLc was mixed although AFCo2 moved the balance toward the induction of IgG2a (IgG2a/IgG1 = 1.04) and PLc toward IgG1 (IgG2a/IgG1 = 0.89) production. In addition, the anti-PLc IgA in saliva and faeces were significantly higher in mice immunised intranasally with AFCo2 (p<0.05) than in mice immunised with PLc via the same route.

**Table 2:** Systemic and mucosal immune responses induced by mucosal immunization of AFCo2 or PLc in BALB/c mice.

Treatment Groups	IgA		IgG sera	IgG2a/IgG1	Vibriocidal activity
	IgA saliva	IgA faeces			
PLc (i.g)	-	-	-	-	-
AFCo2 (i.g)	0.48±0.12^c^	0.55±0.15^b^	-	-	-
Placebo (i.g)	-	-	-	-	-
PLc (i.n)	0.65±0.09^b^	0.44±0.16^b^	2.9±0.14^a^	0.89	2.0±0.3^a^
AFCo2 (i.n)	0.95±0.18^a^	0.72±0.11^a^	3.1±0.09^a^	1.04	2.3±0.4^a^
Placebo (i.n)	-	-	-	-	-

AFCo2 or PLc (100 µg) was administered by i.n or i.g route in BALB/c mice. Results of anti-PLc IgA in saliva and faeces and anti-PLc IgG in sera are expressed as the logarithm of the mean ± SEM. The ratio of IgG subclasses IgG2a/IgG1 is also reported. Vibriocidal activity of sera from immunized mice is also expressed as logarithm of the mean ± SEM. Statistical significance of the variance between multiple groups of experiments were calculated with one-way ANOVA, followed by a Tukey's multiple comparison test.

a,b,c Different letters on the same column mean that values are significantly different (p<0.05); - no response detected.

### Evaluation of the immune response induced by i.n administration of AFCo2 or PLc with OVA

OVA was admixed with AFCo2 (AFCo2 plus OVA) or PLc (PLc plus OVA) and administered to BALB/c mice via the i.n route. The effect of co-administration was evaluated taking into consideration the anti-OVA IgA and IgG, as well as IgG subclass response of specific antibodies elicited in mucosal (saliva and faeces) and systemic (sera) samples, respectively. Table 3 shows that saliva and faeces from mice intranasally immunised with AFCo2 plus OVA had significantly (p<0.05) higher anti-OVA IgA titres than from mice immunised only with OVA or PLc plus OVA. Although, PLc plus OVA induced a significantly (p<0.05) higher mucosal immune response (anti-OVA IgA in saliva and faeces) compared with the response from mice immunised only with OVA, anti-OVA IgG responses in mice immunised with AFCo2 or PLc plus OVA were not statistically significant (p>0.05). A mixed IgG2a/IgG1 response was observed in both treatments (Table 3).

**Table 3:** Systemic and mucosal anti-OVA IgA and IgG response induced by intranasal immunization of AFCo2+ OVA or PLc+OVA in BALB/c mice.

Treatment Groups	IgA		IgG sera	IgG2a/IgG1
	IgA saliva	IgA faeces		
AFCo2+OVA	0.93±0.12^a^	0.78±0.16^a^	2.01±0.18^a^	0.95
PLc+OVA	0.74±0.14^b^	0.44±0.14^b^	2.12±0.2^a^	0.78
OVA	0.19±0.08^c^	0.15±0.07^c^	-	-
Placebo	-	-	-	-

AFCo2 or PLc (100 µg) was admixed with 20 µg of OVA and administered by i.n route to BALB/c mice. Results are expressed as the logarithm of the mean ± SEM. Statistical significance of the variance between multiple groups of experiments were calculated with one-way ANOVA, followed by a Tukey's multiple comparison test.

a,b,c Different letters on the same column mean that values are significantly different (p<0.05); - no response detected.

## Discussion

Mucosal vaccination is considered an appropriate strategy to combat emerging and re-emerging infectious diseases because of the ability to induce both mucosal and systemic immune responses [8]. Various strategies have been employed to improve immunogenicity and delivery of antigens to mucosal surfaces [9]–[10]. PLc and AFCo2 are particulate structures derived from *V. cholerae* O1 with adjuvant and vaccine potential, although AFCo2 are more immunogenic at the mucosal level than PLc when administered by the intranasal route [6]. Different assemblies of antigens, immunostimulators and components of both structures may be responsible for this effect. AFCo2 have a micro-tubular structure whereas PLc are nano proteoliposomes. AFCo2 have been described as cochleates due to light microscopy observations [6]. However more evidence is needed to affirm this from a physical and morphological point of view [1]–[3]. We have reported here that the nanosized PLc may be transformed into tubular microparticles of 16.3±4.6 µm in length (AFCo2) in a calcium environment. AFCo2 form a milky white suspension, when dissolved in a detergent buffer, which is presumably the result of calcium interaction with the negative components of the PLc. Measurements of the length of the structures observed by TEM correspond with previous results [6] obtained by light microscopy and reveals that the width of the AFCo2 are in the range of 2 to 4.5 µm (Figures 2B and 2C); this is much larger than the size reported for cochleates obtained using “pure or synthetic lipids” [1]–[2]. The rotary dialysis method performed involves the removal of detergent against a calcium chloride solution and the transformation mechanism may be explained by competition between removal of detergent from the detergent/phospholipids/drug micelles and the condensation of bilayers by calcium [2]. Some research has been performed to observe the interactions that occur in cochleates using synthetic or purified lipids [1]–[3]. However, cochleates obtained from bacterial proteoliposomes have additional interactions resulting from the presence of LPS and protein antigens that may have impact on the supramolecular organization of the structure [4]. In particular, this may arise from OmpU porin, a representative protein in PLc which has an isoelectric point of 4.2 [6] and a negative charge at the neutral pH (pH 7.4) at which AFCo2 is prepared. Additionally, the outer membrane of *V. cholerae* contains negatively charged phospholipids [11]–[12] that also point to PLc containing these types of structures.

TEM analysis revealed that negative staining penetrates into the AFCo2, whereas PLc remained impermeable to vanadate (Figures 2A and 2B). This may be explained by observations made through SEM, since in Figure 3A, AFCo2 are presented as multilamellar structures with porous edges (Figure 3B) that allowed negative stain and also larger macromolecules like OVA-TR (>40 kDa) to enter. Fluorescence studies demonstrated that a fraction of OVA-TR is incorporated inside the AFCo2 structure after mixing. Epi-fluorescence studies showed that sonication followed by a wash step did not eliminate OVA-TR associated with the AFCo2 (Figure 5), suggesting that binding had occurred. In addition, a considerable amount of the probe may have been internalized into the AFCo2 structures, because in contrast to undisrupted AFCo2, a higher signal intensity came from the exposed section of the cochleates. Furthermore, loading experiments confirmed that a high amount of labelled OVA was entrapped within the AFCo2 and conversely, OVA-TR was not observed in association with PLc (Figure 5D).

Several authors have proposed and demonstrated that cochleates are compact structures and incorporation of drugs and biological molecules occurs mainly during their formation [2]–[4]. However, AFCo2 derived from bacteria as well as AFCo1 [4] are permeable to small molecules used to stain the samples, and also to bigger molecules. Proteoliposome-derived cochleates, like AFCo1, have been used as adjuvants with heterologous co-administered antigens such as ovalbumin (OVA) [5]. The ultimate goal in producing particulate adjuvants from *V. cholerae* O1 is to construct a delivery system for antigens from other enteric pathogens in order to develop a multiple vaccine candidate. Pérez *et al.* [5] have observed that incorporation of OVA in Co from *N. meningitidis* during the dialysis process or admixture of antigen with the ready-made AFCo1 was feasible, and did not affect the immune response induced against OVA. However, to date no studies have been performed to evaluate the nature of interactions between AFCo1 and OVA. The capacity to load adjuvant-particles like AFCo2 or AFCo1 by simple admixing with antigen may be significant in epidemic settings where speed of vaccine manufacture is critical to minimize resultant mortality and/or morbidity [8]. Further studies are required to establish the antigen relationship with AFCo2 or AFCo1 adjuvants and any advantages that this may confer in inducing immunogenicity. However, the complexity of the bacterial derived structures demand a greater understanding of their composition and the rules leading to the assembly of components, since this may provide information for improved antigen loading efficiency.

FFEM confirms the multilayered nature (Figure 4A) of the AFCo2 structure, but also the spiral formation, characteristic of cochleates. Figure 4B shows the curvature of overlapping layers in AFCo2, but it is difficult to identify a continuous bilayer forming the structure. The cochleate phase represents by definition a single bilayer curvature in only one direction [1]–[3]. Meyer *et al.* [13] demonstrated that phosphatidylcholine/Ca cochleates alternate convex-concave bilayer curvatures after cold-hydration. Therefore, AFCo2 does not have the typical folding observed in previous work [1]–[3], [13]. However other authors have found that both curved structures may be interrelated and convex-concave bilayer deformations are observed after cochleate formation [14]. In addition, Marone *et al.* [15] have described different supramolecular structures formed as a result of different drug-, phospholipid- or cation-interactions during cochleate formation; they showed micrographs of cochleates with multilamellar rolled 1–5 µm cylinders in 1,2-dioleoyl-sn-Glycero-3-[Phospho-L-Serine], spherical indented rolled 200–300 nm particles with Soy phosphatidylserine and planar 1 µm multilaminar sheets in palmitoyl-oleoyl phosphatidylserine. These authors also found that treatment with different cations such as calcium, magnesium, zinc, or barium rendered different structural organization where continuity of layers and also tubular shape was lost.

Another observation from the TEM results showed that smaller vesicles where identified in the same preparation with the AFCo2. Such findings are not new, and others have reported that liposomes may coexist with cochleate structures obtained from pure lipids [2]–[3], [15], but in this context it appears as though the vesicles are formed from the larger AFCo2 probably as a result of the staining treatment and unfolding of the structure (Figure 2C). To provide some understanding for this observation, we devised an *in vitro* experiment (Figure 6 and Table 1) whereby AFCo2 cochleates were unfolded using EDTA (a calcium chelating agent). The process was recorded and Video S1 is available as supporting information. A white milky suspension (AFCo2) was transformed into an opalescent colloidal solution consisting of vesicles of 503±8.0 nm of size. This result emphasizes the importance of calcium in the folding of AFCo2 into a cochleate structure, which is very different to other elongated tubular structures, which are not affected by the presence of the divalent cation [3].

Interestingly, the size of the re-folded vesicles (503±8.0 nm) is higher than the original structure (160.7±1.6 nm). This may be due to some of the DOC detergent, used to re-suspend the proteoliposomes during the cochleate preparation step, being simultaneously entrapped. DOC from unfolded Co may become incorporated into the vesicle membranes to increase their size and may also be responsible for improving the immune response. Barret *et al.* [16] demonstrated the adjuvant effect of DOC on membrane protein antigen from HIV and with known absorption enhancing properties may also increase the *in vivo* penetration of antigens through the mucosal surface [17]. This could explain why AFCo2 was more immunogenic than PLc when administered mucosally (Table 2). However, the IgG2a/IgG1 induced by AFCo2 showed to a bias towards IgG2a production, suggesting a preferential induction of a Th1 type of immune response. This response may be related to the difference in the size of both structures. Particles exceeding 5 µm are considered optimal for nasal application [18] and better retained within Peyer's patches [19]; anything smaller than this will travel to the lymphatics. This suggests that the size of the antigen can determine the nature of an immune response and that larger antigens held within local lymph nodes may stimulate local mucosal immune responses while smaller antigens, escaping to the peripheral lymphatics will induce better systemic immune responses or tolerance [19]–[20]. We have also shown that the size of lipid vesicles plays an important role in Th1/Th2 bias in intragastric administration whereby particles below a Z average of 250 nm induced a Th2 response and above a Z average of 980 nm resulted in a Th1 response [21]. This effect has been shown systemically to be a direct result of how small and large lipid particles are trafficked to antigen presenting cell late endosomes and early endosomes, respectively [22]. AFCo2 also demonstrate to have an adjuvant effect to intranasally coadministered OVA at systemic and mucosal as well as affecting the IgG2a/IgG1 ratio (Table 3). Then immunepotentiator and delivery potentialities of cochleates derived from microorganism [5] are also observed in AFCo2. Therefore, this study provides further evidence for the influence on Th1/Th2 responses based on lipid particle size used to present antigen.

## Conclusions

Microscopic characterization disclosed that AFCo2 adopted multilamellar, cochleate-like structures with the classic “spiral-cochleate” pattern of overlapping layers, although no continuous bilayers could be identified. Unlike previous studies we demonstrated that the cochleate-like morphology of AFCo2 had aqueous spaces within the structure that could accommodate additional antigens and small molecules. AFCo2 were immunogenic following intragastric administration and enhanced the immune response to co-administered heterologous antigens when administered intranasally. Therefore, proteoliposome derived cochleate-like structures such as AFCo2 provide potential to be a platform adjuvant technology for the design of a wide range of multivalent mucosal vaccine formulations. However, although AFCo2 is an efficient immune activator, additional characterization is still required before regulatory and commercial adoption. These include standardization of adjuvant composition in particular the LPS, outer membrane proteins and lipopeptide components of the adjuvant derived from the microorganism, known to be ligands for toll-like receptors and powerful activators of the immune system. Nevertheless, since proteoliposomal and cochleate based formulations have been used parenterally, with low toxicity, we expect mucosal formulations to be equally viable. In addition, Perez *et al.* demonstrated in 2006 [23] that a dose of 100 µg of PLc has less than 3000 endotoxin units (EU) and the Neisseria proteoliposome is approved for human use with less than 10 000 EU per dose by the parenteral route. The next stages of reproducible manufacturing techniques including fermentation, extraction, purification and processing will be critical in realising a product with suitable attributes for regulatory approval. Work is presently continuing in evaluating the importance of each of these parameters in adjuvant optimisation. In parallel we are also exploring the incorporation of heterologous and immunologically relevant antigens such as Polysaccharide Vi of *Salmonella* Typhi in order to design a multivalent vaccine against common enteric pathogens that offers an alternative to the killed whole cell or live vaccines that are currently available.

## Supporting Information

Video S1 **AFCo2 unfolding with EDTA.** This video shows the unfolding process of AFCo2 under the action of EDTA chelating agent (25 mM). AFCo2 starts to shrinks in the first 15 seconds and completely disappears at 25 seconds of exposure with EDTA.
(AVI)
